# Epidemiological Study on Metal Pollution of Ningbo in China

**DOI:** 10.3390/ijerph15030424

**Published:** 2018-02-28

**Authors:** Zhou Li, Hong Su, Li Wang, Danbiao Hu, Lijun Zhang, Jian Fang, Micong Jin, Samuel Selorm Fiati Kenston, Xin Song, Hongbo Shi, Jinshun Zhao, Guochuan Mao

**Affiliations:** 1 Department of Preventative Medicine, Zhejiang Key Laboratory of Pathophysiology, Medicine School of Ningbo University, 818 Fenghua Road, Ningbo 315211, China; lizhou0601@163.com (Z.L.); suhong0908@126.com (H.S.); newsamsel@yahoo.com (S.S.F.K.); songxin3c@163.com (X.S.); 2 Ningbo Centers for Disease Prevention and Control, Ningbo 315010, China; ningbowl@163.com (L.W.); jcmjc@163.com (M.J.); shihb@nbcdc.org.cn (H.S.); 3 Ninghai Centers for Disease Prevention and Control, Ninghai 315600, China; hudanbiao@163.com; 4 Jiangdong District Centers for Disease Control and Prevention, Ningbo 315040, China; zljnb@126.com; 5 Zhenhai District Maternal and Child Health Family Planning Center, Ningbo 315200, China; Fjian01@163.com

**Keywords:** metal pollution, vegetable, rice, irrigation water, human hair

## Abstract

*Background*: In order to search for effective control and prevention measures, the status of metal pollution in Ningbo, China was investigated. *Methods*: Nine of the most common contaminating metals including lead (Pb), cadmium (Cd), copper (Cu), iron (Fe), manganese (Mn), chromium (Cr), nickel (Ni), zinc (Zn), and mercury (Hg) in samples of vegetables, rice, soil, irrigation water, and human hair were detected using inductively coupled plasma-mass spectrometry (ICP-MS). Three different districts including industrial, suburban and rural areas in Ningbo were studied through a stratified random sample method. *Results*: (1) Among all of the detected vegetable samples, Cd exceeded the standard limit rates in industrial, suburban and rural areas as high as 43.9%, 27.5% and 5.0%, respectively; indicating the severity of Cd pollution in Ningbo. (2) The pollution index (PI) of Cd and Zn in soil (1.069, 1.584, respectively) suggests that soil is slightly polluted by Cd and Zn. Among all samples, metal contamination levels in soil were all relatively high. (3) A positive correlation was found between the concentrations of Pb, Cd and Cu in vegetables and soil; Pb, Cu, Cr and Ni in vegetables and irrigation water, as well as, Cu and Ni in rice and irrigation water; and, (4) Higher Pb and Cd concentrations were found in student scalp hair in both industrial and suburban areas compared to rural areas. (5) Hg and Pb that are found in human scalp hair may be more easily absorbed from food than any of the other metals. *Conclusions*: In general, certain harmful metal pollutions were detected in both industrial and suburban areas of Ningbo in China.

## 1. Introduction

Rapid industrialization in China has resulted in serious environmental pollution. Among the different types of pollutions, metal pollution is considered to be one of the major environmental issues [1]. Numerous applications of pesticides and fertilizers, industrial waste, mining, and smelting processing have drastically altered the balance and biogeochemical cycles of metals in the environment [2]. During the industrial processes, especially in the industrial and suburban areas, wastewater and solid waste may bring metals into the soil and water bodies [3]. Metals in the soil may then be transferred into crops or vegetables [4]. Metals in the water can also accumulate in the aquatic plants and aquatic animals [5,6]. Metals adhered to the industrial waste particles may suspend and travel in the air, and eventually enter the human body through different pathways [7,8].

After entering the human body, it is difficult for metals to be completely metabolized. They may accumulate in soft tissues or in the bones, causing toxic effects [9,10,11]. In the human body, metals can insert adverse effects on cellular organelles and components, as well as enzymes involved in metabolism, detoxification, and DNA damage repair [12]. Recent studies show that lead can even result in a reduction in intelligence quotient (IQ) [13] and an increase of behavioral abnormities [14]. Mercury can accumulate in brain tissue and cause neurological dysfunction in humans and animals [15]. In addition, cadmium, hexavalent chromium and nickel compounds have been classified as human carcinogens by The International Agency for Research on Cancer (IARC). In the past decade, the concentration of metals in the soil of China was reported to be higher than normal and the detectable concentration of metals in certain foods was found to exceed the health standard limits [16] drawing great public attention and outcry [17]. As a major port city in China, Ningbo is known for the variety of aquatic products it offers. The health hazards of metal pollution through fish consumption have attracted wide attention in recent years [18]. As an important coastal economic district, Ningbo is one of the most industrialized cities in eastern China, however, few studies have been conducted to investigate the status of the metal pollution of this city. Exploring the pollution status of metals will be important for evaluating their potential health risks [19].

Therefore, the following objectives are included in the present study: (1) investigate the status of the nine most common contaminating metals, including lead (Pb), cadmium (Cd), copper (Cu), iron (Fe), manganese (Mn), chromium (Cr), nickel (Ni), zinc (Zn), and mercury (Hg) from industrial, suburban and rural areas in Ningbo; (2) analyze the relationship between concentrations of selected metals in vegetables, rice, soil, irrigation water, and student scalp hair and search for an effective way to control and prevent these environmental pollutions; and, (3) provide some insights into metals accumulation in the agricultural ecosystem and human tissue to serve as a basis for comparison to other regions both in China and worldwide.

## 2. Materials and Methods

### 2.1. Study Area and Sampling

The Ningbo area is an important coastal industrial city which is located on a typical flat alluvial plain in the Yangtze River Delta region (28°510′–30°330′ N, 120°550′–122°160′ E) [19]. Because of increased industrial development and rapid urban expansion in the past several decades, the soil environment is faced with metal contamination due to increasing pollutant inputs from anthropogenic sources. It is characterized as a subtropical mild and humid climate, a mean annual temperature of 16.4 °C, and an annual precipitation of 1480 mm. Due to rapid industrialization, metal pollution in paddy fields in the study area is of increasing concern. Moreover, the area is an important chemical industrial base in China. The textile and garment, chemical, and machinery industries are three main industrial pillars. Petrochemical, electronic, metallurgy, engineering, and building materials have also been developed [19]. In addition, Zhoushan Fisheries in Ningbo with rich fishing, has a large number of beach breeding base. The consumption of seafood may be one of sources of several kind of metals.

Three different districts including industrial, suburban and rural areas in Ningbo were studied by a stratified random sample method. The industrial and suburban areas were assumed to be possible severe contaminated areas, and the rural area was set as possibly none or a slightly contaminated area. The downtown area was not included in this study due to the uncertainty where the consumed vegetables or rice was produced, but the industrial and suburban areas are normally consumed from local produced vegetables and rice. One town or one street in each area was selected for sampling (where there was no electroplating, batteries, metal smelting, leather or other serious metal factories found within 0.5 km of the industrial area). Ten samples of greens, radish, cabbage, celery, and soil were collected from the farmlands located around the residential houses. The collected rice samples were produced in the local farmland, as well as, water samples used to irrigate these farmlands. Rice samples were not collected from suburban areas, because most residents in the suburban area purchase rice from other provinces or abroad. Hair samples were obtained from the local school students who had stayed in the sampling regions for more than five years. A total of 399 students (214 girls and 185 boys, 5–14 years old) were selected to participate in this study, (136 from rural areas, 161 from industrial areas, and the remaining 102 from suburban areas). All of the students were given an informed consent (written) for inclusion before they participated in the study. The study was conducted in accordance with the Declaration of Helsinki, and the protocol was approved by the Ethics Committee of the Ningbo University Ethics Committee (No. AEWC-2013-115) on 19 June 2013. Only the first 2.0 cm of hair proximal to the scalp was used for analysis [20].

### 2.2. Metal Detection

Soil samples (approximately 500 g) were collected from the farmland surface (10–20 cm deep) and air-dried in the laboratory. Soil samples (0.25 g) were digested with a 6 mL acid mixture of 1:1:1 HNO_3_-HCl-HF in a test tube [21]. The mixture was stirred and heated under reflux at 120 °C for 1 h, and then diluted to 50 mL with de-ionized water. Human hair samples were washed three times in de-ionized water and then dried on clean paper. Then, 0.5 g of hair sample was digested in a mixture of 5:1:2 HNO_3_-H_2_O_2_-H_2_O at 130 °C until no residue remained in the solution. The digested samples were cooled at room temperature and diluted to 25 mL with de-ionized distilled water for further analysis. Food samples were oven-dried at 105 °C for 1 h and then at 70 °C until constant weight. Then, food samples were comminuted using a pulverizer and sieved through a nylon sieve with 100 meshes. Approximately 0.2 g food powder was digested by using 3 mL HNO_3_ at 100 ± 5 °C until no residue remained. All of the samples were treated and analyzed according to the national standard method GB/T5009-2003 [22]. All the samples were collected and treated fresh and detected by inductively coupled plasma-mass spectrometry (ICP-MS, Perkin-Elmer NexION 300D, PerkinElmer, Massachusetts, MA, USA) [22]. The accuracy of the determinations was verified using Chinese standardized reference materials: GBW10014 for GSB-5 cabbage, GBW10015 for GSB-6 green, GBW10010 for GSB-1 rice, GBW07424 for GSS-10 soil. The samples were measured in duplicate. For these procedures, reagent blanks were used in the analysis for quality assurance and quality control showed good precision throughout. The national hygienic standard for food and class II standard for soil in China is shown in Table 1. Chemical reagents used in this study were all ultrahigh purity [23].

### 2.3. Food Samples and Environmental Samples of Metal Pollution Assessment

The food samples included 120 vegetable samples and 20 rice simples, the environmental samples included 30 soil samples and 30 irrigation water samples. The pollution levels of metals in food and environmental samples were evaluated using the pollution index (PI), (Equation (1)) [19];

(1) $$\mathrm{PI}=\frac{C_{i}}{S_{i}}$$

where *C_i_* is the metal concentration of the sample, and *S_i_* is the pollution threshold of *i*, the equation applies for the chemical that was given the national hygienic standard.

### 2.4. The Bioaccumulation Factor (BAF) of Food Samples, Environmental Samples and Student Hair Samples

The bioaccumulation factor (BAF) of each metal was used to assess the transfer of metals from food and environment to hair. It can be calculated as:

(2) $${\mathrm{BAF}}_{1}=\frac{C_{h}}{C_{e}}$$

where *C_h_* and *C_e_* are the total metal concentrations in hair samples and corresponding food and environmental samples, respectively [24].

### 2.5. Statistical Analysis

Statistical analysis was performed using SPSS V17.0 software (International Business Machines Corporation, Nork York, NY, USA) for Windows. Data of metal of food and environment are normal distribution, hair samples are log-normal distribution (One-Sample Kolmogorov-Smirnov Test), the median and percentile were used in hair samples data as statistics description. ANOVA Dunnett, *t*-test and correlation analysis were used. The statistical significance levels were set at *p* < 0.001, *p* < 0.01 and *p* < 0.05.

## 3. Results

### 3.1. Metal Detection in the Food Samples and Environmental Samples

#### 3.1.1. In Food Samples (Vegetables and Rice)

Table 2 shows the metal concentrations in different vegetables. The results indicate that average concentrations of Cd (*q*’ = 0.038, *p* = 0.01), Cu (*q*’ = 0.611, *p* = 0.002), Cr (*q*’ = 0.046, *p <* 0.001), and Ni (*q*’ = 0.066, *p* = 0.007) contained in four kinds of vegetables in the suburban area were significantly higher than that of the rural area; whereas, the average concentrations of Pb (*q*’ = 0.031, *p* = 0.027), Cr (*q*’ = 0.039, *p* = 0.002), Ni (*q*’ = 0.067, *p* = 0.033), and Hg (*q*’ = 0.005, *p* = 0.013) in the industrial area were significantly higher than that of the rural area.

In the greens, the concentrations of Cd (*q*’ = 0.016, *p* = 0.017) and Cr (*q*’ = 0.044, *p* = 0.024) in the suburban area were significantly higher than that of the rural area.

In the radish, the concentrations of Mn (*q*’ = 1.992, *p* = 0.002) in the industrial area and Cd (*q*’ = 0.013, *p* = 0.042) and Cu (*q*’ = 0.328, *p* = 0.012) in the suburban area were significantly higher than that of the rural area.

In the cabbage, only the concentration of Hg (*q*’ = 0.001, *p* = 0.045) in the industrial area was significantly higher than that of the rural area.

In the celery, the concentrations of Pb (*q*’ = 0.074, *p* = 0.046), Cu (*q*’ = 0.494, *p* = 0.029) and Cr (*q*’ = 0.055, *p* = 0.006) in the industrial area and the concentrations of Cu (*q*’ = 0.589, *p* = 0.009) and Cr (*q*’ = 0.055, *p* = 0.006) in the suburban area were significantly higher than that of the rural area.

According to the GB18406.1-2001 standard [25], the level of Cd in the four vegetables that were studied in the suburban, industrial and rural area samples were 43.9%, 27.5%, and 5.0%, respectively exceeding the standard limit (data not shown).

Table 3 shows the metal concentrations in rice. The concentrations of Cu (*t* = 5.078, *p* < 0.001), Cr (*t* = 2.419, *p* = 0.030) and Ni (*t* = 5.078, *p* < 0.001) in the rice in the industrial area were significantly higher, whereas Zn (*t* = −5.728, *p* < 0.001) was significantly lower, than that of the rural area.

#### 3.1.2. In the Environmental Samples (Soil and Irrigation Water)

Table 4 shows the metal concentrations in soil. The mean concentrations of Pb, Cd, Cu, Fe, Mn, Cr, Ni, Zn and Hg in soils are 50.06, 0.29, 53.39, 32,231.12, 733.46, 81.91, 39.53, 396.04, 0.11, and 11.46 mg/kg, respectively. The total Cd in Ningbo area were 32.25% exceeding the standard limit exceed their class II standard. The Hg concentrations in both suburban (*q*’ = 0.110, *p* = 0.016) and industrial (*q*’ = 0.104, *p* = 0.020) areas were significantly higher than that of the rural areas.

Table 5 shows the metal concentrations in irrigation water. Differences in Cd, Cu, and Mn concentrations in the irrigation water were observed among three areas. In irrigation water, Cd (*q*’ = 0.053, *p* = 0.041) and Mn (*q*’ = 251.222, *p* < 0.001) in the industrial area and Cu (*q*’ = 6.37, *p* = 0.04) and Mn (*q*’ = 125.162, *p* = 0.005) in the suburban areas were significantly higher than that in the rural areas.

#### 3.1.3. Assessment of Metal Pollution in Food and Environment

The descriptive statistics of the pollution index (PI) of metal concentrations in food and the environment is presented in Table 6. The PI of each of the metals was calculated to assess the pollution degree of different metals in food and the environment, it was classified according to five classes: PI ≤ 1.0 (safe), 1.0 < PI ≤ 2.0 (slight pollution), 2.0 < PI ≤ 3.0 (mild pollution), 3.0 < PI ≤ 5.0 (moderate pollution), and PI > 5.0 (severe pollution). The PIs of all the elements were at the safe level, except Cd and Zn in soil (1.069, 1.584), which means that the soil is slightly polluted. In addition, according to the PI, Cd contamination levels in vegetables, rice, and soil were relatively high. Among all of the samples, soil contamination levels were relatively high. The PIs of soil were in the following decreasing order: Zn (1.584) > Cd (1.069) > Ni (0.791) > Cu (0.534) > Cr (0.410) > Pb (0.167).

#### 3.1.4. Correlation Analysis of Metals between Environmental Samples and Food Samples

Table 7 shows the correlation analysis of metal concentrations in different environmental samples. A positive correlation was found between the concentrations of Pb (*r* = 0.793, *p* < 0.01), Cd (*r* = 0.745, *p* < 0.01) and Cu (*r* = 0.604, *p* < 0.05) in vegetables and soil. Pb (*r* = 0.648, *p* < 0.05), Cu (*r* = 0.967, *p* < 0.001), Cr (*r* = 0.587, *p* < 0.05), and Ni (*r* = 0.804, *p* < 0.001) in vegetables were correlated with that in irrigation water. Cu (*r* = 0.885, *p* < 0.001) and Ni (*r* = 0.796, *p* < 0.01) in rice were correlated with that in irrigation water.

### 3.2. Metal Detection in Student Scalp Hair Samples

#### 3.2.1. Metal Concentrations in Student Scalp Hair Samples

Table 8 shows the metal concentrations in student hair. Hair samples are log-normal distribution (One-Sample Kolmogorov-Smirnov Test), the median and percentile were used in hair samples data as statistics description. There was a striking difference in the concentration of different metals: the concentrations for Pb, Cd, Cu, Fe, Mn, Cr, Ni, Zn, and Hg were 4.581, 0.096, 7.532, 21.025, 3.134, 0.127, 0.313, 240.978, and 0.242 mg/kg. The median concentrations of Pb, Cd, and Hg in human hair were all within the reference concentration range reported previously [26]. After pairwise comparison between the two areas, metal concentrations in scalp hair of students were in the following order: for Pb: (industrial and suburban areas) > rural areas, for Cd: suburban areas > industrial areas > rural areas, for Cu: (rural areas > suburban areas) > industrial areas, for Fe: suburban areas > industrial areas > rural areas, for Mn: (rural, industrial areas) > suburban areas, for Cr (suburban, rural areas) > industrial areas, for Ni: rural areas > (industrial and suburban areas), for Zn: industrial areas > rural areas > suburban areas, for Hg: (rural areas > suburban areas) > industrial areas. After transformed into log-normal distribution of hair metals, ANOVA, Dunnett and correlation analysis were used (Table 8). Compared to rural areas, the concentrations of Pb (*q*’ = 0.296, *p* < 0.001), Cd (*q*’ = 0.154, *p* < 0.001), Fe (*q*’ = 0.662, *p* < 0.001), and Zn (*q*’ = 0.140, *p* < 0.001) in student hair in industrial areas and the concentrations of Pb (*q*’ = 0.366, *p* < 0.001), Cd (*q*’ = 0.333, *p* < 0.001) and Fe (*q*’ = 0.799, *p* < 0.001) in student hair in suburban areas were all significantly high. When compared to rural areas, the concentrations of Cu (*q*’ = 0.081, *p* = 0.001), Cr (*q*’ = 0.189, *p* < 0.001), Ni (*q*’ = 0.326, *p* < 0.001), and Hg (*q*’ = 0.134, *p* < 0.001) in student hair in industrial areas and the concentrations of Mn (*q*’ = 0.144, *p* = 0.001), Ni (*q*’ = 0.340, *p* < 0.001), and Zn (*q*’ = 0.267, *p* < 0.001) in student hair in suburban areas were all significantly low. Furthermore, in order to investigate the correlation between the studied metal concentrations recorded in the scalp hair, the Pearson’s correlation coefficient was calculated and is shown in Table 9. The correlation analysis showed that the six metals (Pb, Cd, Cu, Mn, Cr and Ni) in the hair of the students were positively correlated with each other. In addition, Fe was positively correlated with Pb, Cd, Cu, Mn, and Cr, and there was a positive correlation between Zn and Cu, Mn and Ni, Hg, and Cu, Cr, Ni.

#### 3.2.2. The Bioaccumulation Factor (BAF) of Metals in the Hair, Food and Environmental Samples

The bioaccumulation factor (BAF) of metals in the hair, food, and environmental samples is shown in Table 10. The largest BAF of hair from students consuming vegetables was for Hg (201.3) and Pb (104.2) and the largest BAF of hair from students consuming rice was for Pb (299.7) and Hg (138.6), which indicates that hair may absorb Hg and Pb more easily from food than any of the other metals. While the smallest BAFs of hair from students consuming vegetables and rice were for Mn (0.7 and 0.3, respectively), in addition, Mn was identified as having the lowest accumulation in all of the hair samples studied. Soil had the largest BAF for Hg (4.033) and the smallest for Fe and Cr (0.001 and 0.001). Irrigation water had the largest BAF for Zn (5203) and the smallest for Mn (23), indicating that Hg and Zn were more easily absorbed by the hair via the environment-food-human system, while Fe, Cr, and Mn were the least absorbed by the hair via this system.

## 4. Discussion

In this study, we found that the average concentrations of Cd, Cu, Cr, and Ni in four evaluated vegetables in the suburban area of Ningbo, China were significantly higher than in the rural area. However, in the industrial area, the average concentrations of Pb, Cr, Ni, and Hg were significantly higher than the rural area. The amount of Cd in the four evaluated vegetables in suburban, industrial and rural area samples reached 43.9%, 27.5%, and 5.0%, respectively, exceeding the standard limit. Rice produced in the industrial area had more Cu, Cr, and Ni contamination than that of the rural area. These results indicate that metal pollution in the suburban and industrial areas were more serious than in the rural area, especially Cd pollution, due to the dramatically increased industrial operations and rapid urban expansion in the past three decades in Ningbo. In addition, the relatively high PI of Cd in vegetables also indicated Cd contamination. The dynamic uptake of metals varies at different growth points for different vegetables [27]. Our results also show that the accumulations of different metals in different vegetables or rice are different.

In soil samples, we found only the Hg concentrations in both suburban and industrial areas were significantly higher than in the rural area [28]. Pb, Cd, Cu, Fe, Mn, Cr, and Ni concentrations in the industrial and suburban soil samples were also higher than that of the rural samples although no significant difference was found. The soil environment is faced with metal contamination due to increasing pollutant inputs from anthropogenic sources. In irrigation water, Cd and Mn in the industrial area and Cu and Mn in the suburban area were significantly higher than in the rural area. The pollution index (PI) suggests that soil is slightly polluted with Cd and Zn. In addition, according to the PI, Cd contamination levels in vegetables, rice, and soil were relatively high. After analyzing the relationship of metal concentrations among different environmental and food samples, we found that the concentrations of Pb, Cd, and Cu in vegetables were positively correlated with that of the soil. Pb, Cu, Cr and Ni in vegetables were positively correlated with that in irrigation water. In addition, Cu and Ni in rice were positively correlated with that in irrigation water. These results indicate that metal contaminations in different vegetables or rice may be the result of soil and irrigation water pollution [29]. Previous studies show that vegetables and crops may take up metals when grown in polluted soil and long-term accumulation of metals in soil can consequently cause an increase in metal uptake by vegetables [30,31].

Our results are in agreement with the conclusion of Briki et al. a higher level of metal contaminations exists in suburban and industrial areas than that of rural areas [32], except for Zn. Interestingly, in this study, we found that Zn concentrations in rice, irrigation water, and human hair in the suburban and industrial areas were all significantly lower than that of the rural areas. The reason for this phenomenon is not clear. It may be caused by the imbalance of different metals in the environment.

The biological monitoring of metal pollution for human exposure is of optimal interest for researchers [33]. As a stable matrix for human bio-monitoring [34], human hair has many advantages, such as easy collection, low cost, easy transport and storage [35], and less invasive [36]. Monitoring of minor or trace elements in human hair has become an effective method for toxic trace metal exposure in recent years [37,38]. Young students are more vulnerable to metal hazards than other age groups and can reflect the environmental influence sensitivity [39,40].

In our study, though median concentrations of Pb, Cd, and Hg in student hair were all within the reference concentration range reported previously, the concentrations of Pb, Cd in industrial and suburban areas were all significantly higher than rural areas. The bioaccumulation factor (BAF) of Pb and Hg in the hair samples from vegetables, rice was relatively high, reflecting that metals accumulated in the students’ bodies mainly originated from food. Soil had the largest BAF for Hg, indicating that Hg were more easily absorbed by the hair *via* the environment-food-human system. Eliminating metals in the environment is the basic method to reduce metal levels in the hair. The correlation analysis showed that the six metals (Pb, Cd, Cu, Mn, Cr, and Ni) in the hair of the students were positively correlated with each other. It can be seen from a correlation among various trace metals that there exists a mutual dependence of these metals in the environment, which is probably due to their similar chemical nature [31]. Many of these associations are difficult to explain with the information available and further studies are required for a better understanding of correlations between different metals.

Pb, Cd, and Hg are nonessential elements, which are widely distributed at low levels in the environment [41], In this study, we found that the average concentrations of Pb and Cd in student hair, including both industrial and suburban areas, were significantly higher than that of rural areas and the Hg concentrations in both suburban and industrial areas were significantly higher than in the rural areas. These results suggest that adverse health effects of harmful metal pollution in the industrial and suburban areas in Ningbo, China should not be neglected [42].

Further studies should focus on the following issues:

(1) More studies concerning the relationship between metals concentrations in human tissue and food should be conducted. (2) More reference of metals in body tissue should be studied and updated frequently, especially for children. (3) Every element in the food chain should be used to investigate the dynamic uptake of metals.

## 5. Conclusions

In general, we found that metal pollution in the suburban and industrial areas was more serious than that in the rural areas; among all the detected vegetable samples, Cd rates exceeds the standard limit and is higher in the industrial and suburban areas when compared to the rural areas. Thus, Cd pollution in Ningbo is a serious one; Higher Pb and Cd concentrations were found in student hair in both industrial and suburban areas compared to rural areas. In conclusion, certain harmful metal pollutions were detected in both industrial and suburban areas of Ningbo in China.

## Figures and Tables

**Table 1:** The national hygienic standard for food and class II standard for soil in China (mg/kg).

National Standard	Samples	Pb	Cd	Cu	Cr	Ni	Zn	Hg
GB18406.1-2001	vegetables	0.2	0.05	-	0.5	-	-	0.01
GB2762-2012	rice	0.2	0.2	-	1	-	-	0.02
GB15618-1995 *	soil	≤300	≤0.30	≤100	≤200	≤50	≤250	≤0.50
GB5084-2005	irrigation water	≤0.20	≤0.01	≤1.00	≤0.10	-	≤2.00	≤0.001

Note: * Class II standard for soil in China.

**Table 2:** Metal concentrations in different vegetables.

Sample	Area	n	Metal Concentrations (mg/kg, Means ± SD)								
			Pb	Cd	Cu	Fe	Mn	Cr	Ni	Zn	Hg
Greens	Rural	10	0.031 ± 0.009	0.026 ± 0.012	0.372 ± 0.114	10.612 ± 5.495	6.851 ± 6.647	0.046 ± 0.015	0.092 ± 0.038	5.978 ± 3.063	0.0014 ± 0.0007
	Industrial	10	0.053 ± 0.058	0.032 ± 0.017	0.577 ± 0.242	7.542 ± 5.359	9.039 ± 8.666	0.043 ± 0.019	0.278 ± 0.332	4.507 ± 2.148	0.0013 ± 0.0006
	Suburban	10	0.062 ± 0.077	0.069 ± 0.056 *	1.579 ± 2.491	9.152 ± 6.873	5.043 ± 3.344	0.090 ± 0.059 *	0.375 ± 0.709	12.07 ± 25.88	0.0012 ± 0.0007
Radish	Rural	10	0.015 ± 0.019	0.006 ± 0.004	0.202 ± 0.099	3.353 ± 3.772	1.001 ± 0.681	0.031 ± 0.010	0.059 ± 0.050	2.623 ± 1.498	0.0007 ± 0.0004
	Industrial	10	0.018 ± 0.016	0.013 ± 0.007	0.267 ± 0.105	3.109 ± 2.878	2.993 ± 1.950 **	0.080 ± 0.087	0.161 ± 0.177	3.965 ± 3.437	0.0013 ± 0.0013
	Suburban	10	0.020 ± 0.020	0.019 ± 0.019 *	0.530 ± 0.403 *	2.461 ± 1.878	0.960 ± 0.503	0.068 ± 0.049	0.224 ± 0.447	3.189 ± 5.932	0.0004 ± 0.0002
Cabbage	Rural	10	0.040 ± 0.039	0.018 ± 0.012	0.254 ± 0.074	11.018 ± 9.212	11.379 ± 17.602	0.041 ± 0.026	0.067 ± 0.029	4.354 ± 3.551	0.0011 ± 0.0005
	Industrial	10	0.067 ± 0.057	0.041 ± 0.039	0.479 ± 0.273	10.488 ± 8.744	8.297 ± 7.892	0.098 ± 0.107	0.261 ± 0.216	6.091 ± 4.199	0.0022 ± 0.0014 *
	Suburban	10	0.072 ± 0.079	0.039 ± 0.022	0.504 ± 0.337	13.152 ± 11.445	2.511 ± 1.203	0.089 ± 0.053	0.208 ± 0.280	6.514 ± 9.435	0.0012 ± 0.0008
Celery	Rural	10	0.018 ± 0.013	0.034 ± 0.017	0.212 ± 0.069	8.182 ± 6.689	3.665 ± 2.552	0.034 ± 0.013	0.030 ± 0.022	2.850 ± 1.340	0.0011 ± 0.0010
	Industrial	10	0.092 ± 0.114 *	0.091 ± 0.118	0.706 ± 0.440 *	11.752 ± 9.377	3.333 ± 2.505	0.089 ± 0.033 **	0.192 ± 0.192	4.407 ± 4.060	0.0018 ± 0.0015
	Suburban	10	0.037 ± 0.038	0.109 ± 0.136	0.801 ± 0.591 **	7.578 ± 3.988	2.186 ± 1.063	0.089 ± 0.055 **	0.225 ± 0.261	3.881 ± 2.556	0.0007 ± 0.0002
Four Vegetables	Rural	40	0.026 ± 0.024	0.021 ± 0.016	0.260 ± 0.111	8.291 ± 7.050	5.724 ± 9.932	0.038 ± 0.017	0.062 ± 0.041	3.951 ± 2.805	0.0011 ± 0.0007
	Industrial	40	0.057 ± 0.073 *	0.044 ± 0.067	0.507 ± 0.323	8.223 ± 7.603	5.916 ± 6.472	0.077 ± 0.072 **	0.223 ± 0.233 *	4.742 ± 3.511	0.0016 ± 0.0013 *
	Suburban	40	0.048 ± 0.061	0.059 ± 0.079 *	0.871 ± 1.376 **	8.112 ± 7.769	2.733 ± 2.403	0.084 ± 0.053 ***	0.261 ± 0.457 **	6.551 ± 14.486	0.0009 ± 0.0006

Note: *** *p* < 0.001, ** *p* < 0.01, * *p* < 0.05. Dunnett test, versus rural.

**Table 3:** Metal concentrations in rice.

Sample Name	Area	n	Metal Concentrations (mg/kg, Means ± SD)								
			Pb	Cd	Cu	Fe	Mn	Cr	Ni	Zn	Hg
Rice	Rural	10	0.027 ± 0.034	0.071 ± 0.061	2.147 ± 0.737	12.46 ± 19.227	11.938 ± 4.33	0.072 ± 0.044	0.154 ± 0.082	15.899 ± 2.581	0.0017 ± 0.0012
	Industrial	10	0.004 ± 0.000	0.132 ± 0.043	8.002 ± 2.530 ***	1.471 ± 0.418	8.704 ± 2.757	0.128 ± 0.042 *	0.519 ± 0.086 ***	8.115 ± 2.359 ***	0.0018 ± 0.0004

Note: *** *p* < 0.001, * *p* < 0.05. *t*-test, versus rural.

**Table 4:** Metal concentrations in soil.

Sample Name	Area	n	Metal Concentrations (mg/kg, Means ± SD)										
			Pb	Cd	Cu	Fe	Mn	Cr	Ni	Zn	Hg
Soil	Rural	10	34.56 ± 6.42	0.22 ± 0.08	31.6 ± 19.02	30,077.0 ± 27,551.0	604.0 ± 255.0	59.7 ± 55.5	34.86 ± 40.06	393.5 ± 558.5	0.03 ± 0.03	
	Industrial	10	57.32 ± 34.10	0.24 ± 0.09	47.73 ± 14.59	31,225.8 ± 13,781.5	992.0 ± 1071.0	100.0 ± 29.5	46.34 ± 10.57	582.9 ± 622.2	0.14 ± 0.08 *	
	Suburban	10	57.56 ± 28.58	0.49 ± 0.51	78.34 ± 94.18	35,103.3 ± 7764.2	616.1 ± 204.3	85.6 ± 34.4	37.59 ± 12.17	228.4 ± 166.0	0.14 ± 0.12 *	

Note: * *p* < 0.05. Dunnett test, versus rural.

**Table 5:** Metal concentrations in irrigation water.

Sample Name	Area	n	Metal Concentrations (μg/L, Means ± SD)								
			Pb	Cd	Cu	Fe	Mn	Cr	Ni	Zn	Hg
Irrigation Water	Rural	10	0.56 ± 0.83	0.04 ± 0.02	0.72 ± 0.29	152.34 ± 112.44	10.37 ± 11.97	0.62 ± 0.22	0.55 ± 0.23	64.13 ± 191.82	0.03 ± 0.02
	Industrial	10	1.70 ± 2.16	0.09 ± 0.08 *	3.99 ± 1.73	190.53 ± 73.04	261.59 ± 136.40 ***	3.09 ± 4.36	10.84 ± 17.73	52.89 ± 67.19	0.04 ± 0.01
	Suburban	10	1.52 ± 0.72	0.05 ± 0.01	7.46 ± 10.55 *	212.67 ± 69.46	135.53 ± 55.73 ***	2.04 ± 0.93	4.21 ± 2.63	21.93 ± 16.32	0.06 ± 0.05

Note: *** *p* < 0.001, * *p* < 0.05. Dunnett test, versus rural.

**Table 6:** Descriptive statistics of PIs metal concentrations in food and the environment.

Samples	Pb	Cd	Cu	Cr	Ni	Zn	Hg
Vegetables	0.220	0.832	-	0.133	-	-	0.012
Rice	0.100	0.450	-	0.089	-	-	0.087
Soil	0.167	1.069	0.534	0.410	0.791	1.584	0.215
Irrigation Water	0.006	0.006	0.004	0.019	-	0.023	0.042

**Table 7:** Correlation analysis of metal concentrations among different environmental samples.

		Vegetables	Rice
Pb	soils	0.793 **	−0.382
	Irrigation water	0.648 *	−0.064
Cd	soils	0.745 **	0.152
	Irrigation water	0.442	0.023
Cu	soils	0.604 *	0.484
	Irrigation water	0.967 ***	0.885 ***
Fe	soils	−0.335	−0.087
	Irrigation water	0.225	−0.206
Mn	soils	−0.218	0.376
	Irrigation water	−0.079	−0.246
Cr	soil	0.339	−0.135
	Irrigation water	0.587 *	0.279
Ni	soils	0.295	0.169
	Irrigation water	0.804 ***	0.796 **
Zn	soils	0.382	−0.555
	Irrigation water	−0.112	−0.026
Hg	soils	−0.088	−0.151
	Irrigation water	0.525	0.530

Note: *** *p* < 0.001, ** *p* < 0.01, * *p* < 0.05, correlation analysis.

**Table 8:** Metal concentrations in student scalp hair in the rural, industrial and suburban areas.

Area	n	Metal Concentrations (mg/kg, Median (Q25, Q75))								
		Pb	Cd	Cu	Fe	Mn	Cr	Ni	Zn	Hg
Rural	136	2.99 (1.87, 4.48)	0.07 (0.05, 0.10)	8.46 (7.00, 10.91)	5.38 (4.50, 7.31)	3.64 (2.29, 5.67)	0.15 (0.10, 0.22)	0.63 (0.36, 1.01)	250.25 (185.60, 376.08)	0.26 (0.20, 0.33)
Industrial	161	5.38 *** (3.33, 9.67)	0.10 *** (0.06, 0.16)	6.67 *** (5.73, 9.13)	24.67 *** (19.87, 35.32)	3.05 (1.95, 5.50)	0.10 *** (0.06, 0.15)	0.27 *** (0.15, 0.45)	331.41 *** (225.15, 578.85)	0.20 *** (0.13, 0.28)
Suburban	102	7.38 *** (4.18, 11.91)	0.14 *** (0.10, 0.21)	7.74 (6.36, 10.67)	34.31 *** (26.60, 47.38)	2.81 *** (1.71, 4.34)	0.16 (0.11, 0.23)	0.22 *** (0.15, 0.37)	145.85 *** (112.32, 179.60)	0.29 (0.17, 0.47)
Total median	399	4.58 (2.73, 7.95)	0.010 (0.06, 0.16)	7.53 (6.21, 10.29)	21.03 (6.91, 33.48)	3.13 (1.94, 5.27)	0.13 (0.09, 0.20)	0.31 (0.18, 0.71)	240.98 (170.46, 400.10)	0.24 (0.16, 0.33)
Published Range [26]		0.004–95	0.02–16	-	-	-	0.03–16	-	-	0.07–106

Note: Note: *** *p* < 0.001. ANOVA, Dunnett, after transformed into log-normal distribution of hair metals.

**Table 9:** Correlation analysis of nine metals in the hair of students.

	Pb	Cd	Cu	Fe	Mn	Cr	Ni	Zn	Hg
Pb	-								
Cd	0.660 ***	-							
Cu	0.346 ***	0.366 ***	-						
Fe	0.555 ***	0.478 ***	0.136 **	-					
Mn	0.470 ***	0.435 ***	0.410 ***	0.163 **	-				
Cr	0.253 ***	0.262 ***	0.322 ***	0.103 *	0.318 ***	-			
Ni	0.195 ***	0.180 ***	0.523 ***	−0.082	0.533 ***	0.336 ***	-		
Zn	0.095	0.010	0.221 ***	−0.026	0.272 ***	0.007	0.384 ***	-	
Hg	−0.010	−0.022	0.163 **	−0.053	−0.039	0.121 *	0.117 *	−0.105 *	-

Note: *** *p* < 0.001, ** *p* < 0.01, * *p* < 0.05, correlation analysis.

**Table 10:** Bioaccumulation factor (BAF) of metals between student’s hair, food and environmental samples.

Ratio	Pb	Cd	Cu	Fe	Mn	Cr	Ni	Zn	Hg
hair/vegetables	104.2	2.3	13.7	2.6	0.7	1.9	1.7	47.3	201.3
hair/rice	229.7	1.1	1.9	2.3	0.3	1.4	1.2	17.9	138.6
hair/soil	0.148	0.533	0.217	0.001	0.004	0.001	0.006	1.865	4.033
hair/irrigation	3636	1655	1856	114	23	66	60	5203	5717
